# Complete Genome Sequence of Carbendazim-Degrading *Mycobacterium* sp. Strain djl-10

**DOI:** 10.1128/genomeA.01683-16

**Published:** 2017-02-23

**Authors:** Ji Zhang, Qiaoyun Yuan, Wei Yang, Xinfeng Wang

**Affiliations:** Jiangsu Collaborative Innovation Center of Regional Modern Agriculture and Environmental Protection, Jiangsu Key Laboratory for Eco-Agricultural Biotechnology around Hongze Lake, School of Life Science, Huaiyin Normal University, Huai’an, People’s Republic of China

## Abstract

*Mycobacterium* sp. strain djl-10, an efficient degrader of carbendazim, was isolated from a carbendazim manufacturing wastewater treatment system. Here, we report the complete genome sequence of djl-10, which consists of a chromosome and three plasmids.

## GENOME ANNOUNCEMENT

Carbendazim is a fungicide widely used around the world to control a broad range of fungal diseases in agricultural crops (1, 2). *Mycobacterium* sp. strain djl-10, a carbendazim-degrading strain, was isolated from a carbendazim manufacturing wastewater treatment system. Our clarification of the complete genome sequence of this strain is expected to provide insights into the mechanisms of carbendazim degradation by the strain and provide help for its application in corresponding carbendazim-contaminated environment remediation in the future.

The complete genome of *Mycobacterium* sp. djl-10 was generated using the Illumina HiSeq and PacBio RS single-molecule sequencing platforms. For this purpose, two libraries (500-bp paired-end library for Illumina HiSeq and 8- to ~10-kb SMRT Bell library for PacBio RS) were constructed. A total of 3,308,392,500 bp of Illumina raw data, 3,132,093,644 bp of Illumina high-quality data, and 134,281,312 bp of PacBio raw data were generated. The sequencing data of Illumina high-quality reads were assembled by SOAP*denovo* (version 2.04) and used to align with PacBio sequencing data by blasR to reduce the single-base and insert-missing errors of long single-molecule sequence. The corrected single-molecule sequencing data of PacBio were connected using the overlap relationship between sequence scaffolds, and then Celera assembler 8.0 was used for subsequent assembly. After completing all the scaffold connection, the Illumina data were used again to verify and close gaps of the assembled scaffolds by GapCloser version 1.12 (SOAP*denovo*-related software) (3).

The genome of djl-10 consists of a circular chromosome of 6,395,946 bp, with 67.93% G+C content, and three plasmids (plasmid1, 134,689 bp; plasmid2, 21,705 bp; plasmid3, 26,982 bp). A total of 46 tRNA genes encompassing all 20 amino acids and two copies of rRNA operons (5S-23S-16S) were identified by using tRNAscan-SE version 1.3.1 (4) and Barrnap 0.4.2 (RNAmmer-1.2) (5), respectively. Genes were predicted by Glimmer 3.02 (http://ccb.jhu.edu/software/glimmer/index.shtml). There were 6,392 genes in total, with a length of 6,120,036 bp with 68.2% G+C content, which accounts for 92.4% of the entire genome. To annotate the genes, all the corresponding protein sequences were aligned in the databases of Nr, genes, string, and GO, by BLASTp (BLAST2.2.28+). Among all the predicted genes, 6,020 genes hit top target sequences and achieved annotation information.

A putative methyl-1H-benzimidazol-2-ylcarbamate (MBC)-hydrolyzing esterase (*mhe*) gene involved in carbendazim utilization located on plasmid1 was found with the CDS of BAC37_RS31035, and it showed 100% and 99% sequence identity with *mheI* genes cloned from *Rhodococcus erythropolis* djl-11 (6) and *Nocardioides* sp. SG-4G (7), respectively. The genes responsible for the subsequent degradation of carbendazim in djl-10 are still unknown and will be clarified in our future work. The availability of the *Mycobacterium* sp. djl-10 genome sequence will act as an invaluable supplement to the ongoing research efforts toward understanding several unanswered questions associated with the degradation of carbendazim and would thus aid in the development of* in situ* bioremediation in the future.

### Accession number(s).

The genome and three plasmid sequences have been deposited in GenBank under accession numbers CP016640, CP016641, CP016642, and CP016643, respectively.
